# Analysis of the vibrational characteristics of diamane nanosheet based on the Kirchhoff plate model and atomistic simulations

**DOI:** 10.1186/s11671-023-03887-5

**Published:** 2023-08-31

**Authors:** Zhuoqun Zheng, Fengyu Deng, Zhu Su, Haifei Zhan, Lifeng Wang

**Affiliations:** 1 State Key Laboratory of Mechanics and Control for Aerospace Structures, Nanjing University of Aeronautics and Astronautics, Nanjing, 210016 China; 2 College of Civil Engineering and Architecture, Zhejiang University, Hangzhou, 310058 China; 3 School of Mechanical, Medical and Process Engineering, Queensland University of Technology, Brisbane, QLD 4001 Australia

**Keywords:** Diamane, Natural frequency, Modal shape, Molecular dynamics simulation, Kirchhoff plate model

## Abstract

Single layer diamond—diamane, has been reported with excellent mechanical properties. In this work, molecular dynamics (MD) simulation and Kirchhoff plate model are utilized to investigate the vibrational characteristics of diamane sheets. The mechanical parameters of diamane sheets, including bending stiffness, Young’s modulus, Poisson’s ratio and coefficient of thermal expansion, are calibrated by using MD simulations. The natural frequencies and corresponding modal shapes of the diamane sheets predicted by the Kirchhoff plate model agree well with that obtained from the MD simulations. It is found that the edges exert marginal effect on the modal shapes when free boundary conditions are applied. Additionally, the Kirchhoff plate model considering the thermal expansion provides reasonable prediction for the natural frequencies of the diamane sheets with all boundary clamped under varying temperatures. This study offers valuable insights into the vibrational properties of diamane sheets, from both a simulation and theoretical standpoint. The findings would be beneficial for the design of nanoscale mechanical resonators utilizing these novel carbon materials.

**Supplementary Information:**

The online version contains supplementary material available at 10.1186/s11671-023-03887-5.

## Introduction

In past years, nanoelectromechanical systems (NEMS) attract interest from broad fields. Lots of appealing applications like the mass spectrometry [1–4], force spectrometry [5–7], and ultrasound vibrations detection [8] are enabled through the ultra-high sensitivity of nanoscale resonators. The excellent vibrational properties of low-dimensional nanomaterials are crucial, which allows the detection of minute frequency shift under external perturbations [9–11]. In the past tens of years, many different low-dimensional nanomaterials have been utilized to make nanoscale mechanical resonators, such as carbon nanotube (CNT) [12], carbon nanothread [13], black phosphorus (BP), graphene sheets (GS), h-BN and MoS_2_ [14]. Recently, the ultrathin 2D diamond films—diamane have been successfully synthesized through *sp*^2^-to-*sp*^3^ transformation of bilayer graphene [15–18]. Previous studies based on molecular dynamics (MD) simulations and density functional theory (DFT) calculations [19–21] have predicted that diamane has excellent thermal and electronic properties. Our previous work [22] shows that the diamane nanoribbon possesses a high merit (defined as natural frequency times quality factor), which make it a good candidate for nanoscale mechanical resonators. However, as a keyway to thoroughly understand the dynamic behaviors of the diamane-based nanoscale mechanical resonators, dynamic models are not given in that work.

The vibration characteristics of nanomaterials play a significant role in the behaviors of nanoscale mechanical resonators. Hence, different low-dimensional nanomaterials have been investigated on their vibrational properties. For instance, the thermally induced vibrations of carbon nanotubes are studied by MD simulations and beam models [23, 24]. Recently, MD simulations as well as beam models are also utilized to investigate the free vibrational properties of ultrafine carbon nanothreads [25]. The natural vibration of single-layered GS [26–28] and single-layered BP [29] are investigated via MD simulations and plate models. Single-layered GS can be treated as the in-plane isotropic material materials, while single-layered BP has anisotropic mechanical properties. Thus, different plate models are developed to predict their vibrational properties. A thin plate model is used for single-layered GS, while an orthotropic plate model is applied for single-layered BP. The free vibrations of circular single-layered MoS_2_ suspended over a circular hole are studied using MD simulations and circular Kirchhoff plate models [30]. For the potential use of diamane as the nanoscale mechanical resonators, it is necessary to have a comprehensive study on the vibrational characteristics of diamane.

In this work, the vibrational properties of diamane are investigated via Kirchhoff plate models and MD simulations. Kirchhoff plate models with modified Fourier series method are developed. The mechanical parameters used in the Kirchhoff plate model are calibrated by MD simulations. Then, boundary conditions influences and temperature influences are discussed respectively. By comparing with the results of MD simulations, some conclusions are summarized.

## Methodology

### Theoretical modelling

**Fig. 1:**
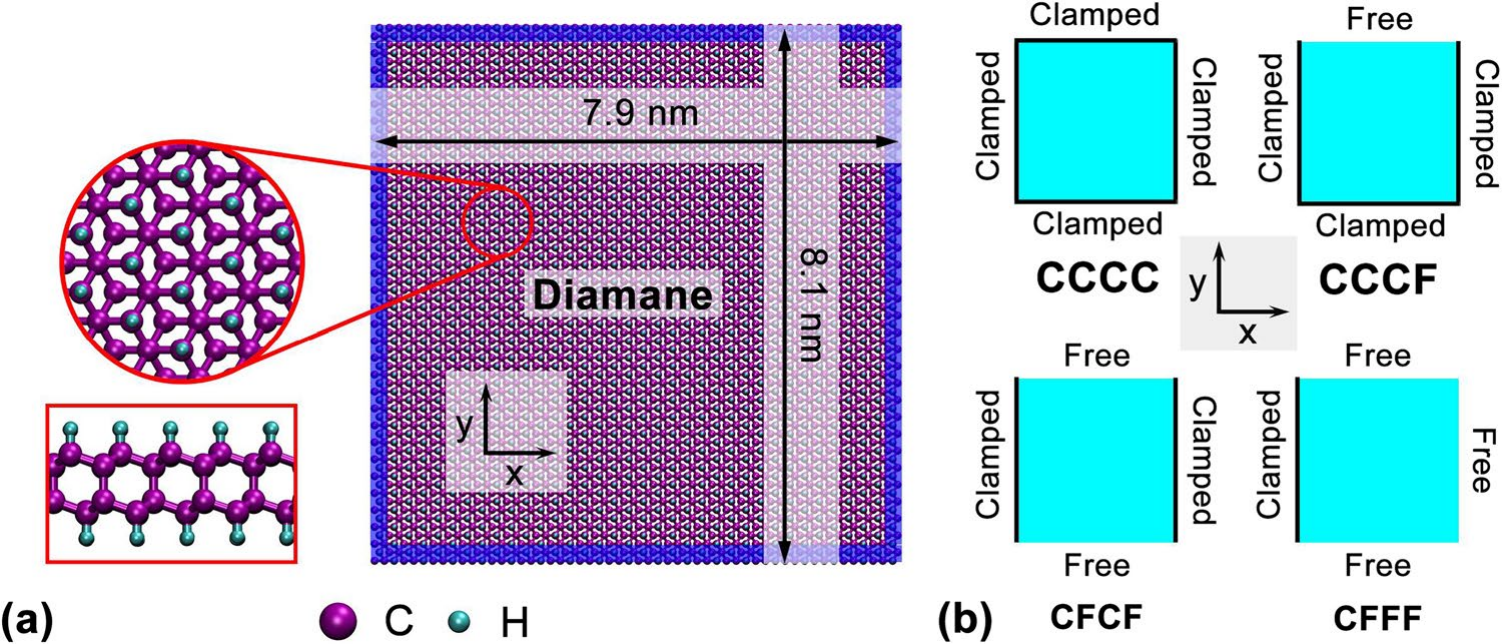
The atomic structure and boundary settings of diamane sheet. **a** Atomic configurations of the diamane sheet. **b** Four different boundary conditions considered in this work

This section deals with the vibrational properties of diamane sheets. Figure 1a shows the atomic structure of the diamane. The right panel is the diamane sheet used in MD simulations, and the left panel is the zoom-in structure. From a theoretical perspective, the diamane sheet is modeled as a rectangle thin plate made of an elastic and in-plane isotropic material. The plate with thickness *h*, mass density *ρ*, the Poisson ratio *μ*, Young’s modulus *E* and the coefficient of thermal expansion *α* is considered under the temperature *T*. The governing equation for the vibration of the plate can be expressed as

1 $$D\nabla^{4} w(x,y,t) + \rho h\frac{{\partial^{2} w(x,y,t)}}{{\partial t^{2} }} + \frac{E\alpha hT}{{1 - \mu }}\nabla^{2} w(x,y,t) = 0$$

where $\nabla^{4} = (\partial^{4} /\partial x^{4} ) + 2(\partial^{4} /\partial x^{2} \partial y^{2} ) + (\partial^{4} /\partial y^{4} )$ and $\nabla^{2} = (\partial^{2} /\partial x^{2} ) + (\partial^{2} /\partial y^{2} )$ are Laplace operators, $D = Eh^{3} /12(1 - \mu^{2} )$ is the bending stiffness of the plate.

As shown in Fig. 1b, four different boundary conditions for the plate are investigated in this work. The boundary conditions with all boundaries clamped (CCCC) are

2a $$w(0,y,t) = w(a,y,t) = w(x,0,t) = w(x,b,t) = 0,$$

2b $$\left. {\frac{\partial w(x,y,t)}{{\partial x}}} \right|_{x = 0} = \left. {\frac{\partial w(x,y,t)}{{\partial x}}} \right|_{x = a} = \left. {\frac{\partial w(x,y,t)}{{\partial y}}} \right|_{y = 0} = \left. {\frac{\partial w(x,y,t)}{{\partial y}}} \right|_{y = b} = 0,$$

The boundary conditions with one side free and other three sides clamped (CCCF) are

3a $$w(0,y,t) = w(a,y,t) = w(x,0,t) = 0,$$

3b $$\left. {\frac{\partial w(x,y,t)}{{\partial x}}} \right|_{x = 0} \left. { = \frac{\partial w(x,y,t)}{{\partial x}}} \right|_{x = a} = \left. {\frac{\partial w(x,y,t)}{{\partial y}}} \right|_{y = 0} = 0,$$

3c $$\left. {\frac{{\partial^{2} w(x,y,t)}}{{\partial y^{2} }}} \right|_{y = b} = 0,$$

3d $$\left. {\frac{{\partial^{3} w(x,y,t)}}{{\partial y^{3} }}} \right|_{y = b} = 0.$$

The boundary conditions with two opposite sides clamped and the other two opposite sides free (CFCF) are

4a $$w(0,y,t) = w(a,y,t) = 0,$$

4b $$\left. {\frac{\partial w(x,y,t)}{{\partial x}}} \right|_{x = 0} = \left. {\frac{\partial w(x,y,t)}{{\partial x}}} \right|_{x = a} = 0,$$

4c $$\left. {\frac{{\partial^{2} w(x,y,t)}}{{\partial y^{2} }}} \right|_{y = 0} = \left. {\frac{{\partial^{2} w(x,y,t)}}{{\partial y^{2} }}} \right|_{y = b} = 0,$$

4d $$\left. {\frac{{\partial^{3} w(x,y,t)}}{{\partial y^{3} }}} \right|_{y = 0} = \left. {\frac{{\partial^{3} w(x,y,t)}}{{\partial y^{3} }}} \right|_{y = b} = 0.$$

The boundary conditions with one side clamped and other three sides free (CFFF) are

5a $$w(0,y,t) = 0,$$

5b $$\left. {\frac{\partial w(x,y,t)}{{\partial x}}} \right|_{x = 0} = 0,$$

5c $$\left. {\frac{{\partial^{2} w(x,y,t)}}{{\partial x^{2} }}} \right|_{x = a} = \left. {\frac{{\partial^{2} w(x,y,t)}}{{\partial y^{2} }}} \right|_{y = 0} = \left. {\frac{{\partial^{2} w(x,y,t)}}{{\partial y^{2} }}} \right|_{y = b} = 0,$$

5d $$\left. {\frac{{\partial^{3} w(x,y,t)}}{{\partial x^{3} }}} \right|_{x = a} = \left. {\frac{{\partial^{3} w(x,y,t)}}{{\partial y^{3} }}} \right|_{y = 0} = \left. {\frac{{\partial^{3} w(x,y,t)}}{{\partial y^{3} }}} \right|_{y = b} = 0.$$

The modified Fourier series method (MFSM) [31, 32] is used to solve Eq. (1). The transverse displacement *w*(*x*,*y*) of the plate can be assumed as

6 $$\begin{aligned} w(x,y) = & \sum\limits_{m = 0}^{\infty } {\sum\limits_{n = 0}^{\infty } {A_{mn} \cos (\lambda_{am} x)\cos (\lambda_{bn} y)} } \\ & + \sum\limits_{l = 1}^{4} {\left( {\sum\limits_{n = 0}^{\infty } {\xi_{b}^{l} (y)c_{m}^{l} \cos (\lambda_{am} x)} + \sum\limits_{n = 0}^{\infty } {\xi_{a}^{l} (x)d_{n}^{l} \cos (\lambda_{bn} y)} } \right)} \\ \end{aligned}$$

where $\lambda_{am} = m\pi /a$, $\lambda_{bn} = n\pi /b$, $\xi_{a}^{l} (x)$ and $\xi_{b}^{l} (y)$ are supplementary functions. Here, $\xi_{a}^{l} (x)$ and $\xi_{b}^{l} (y)$ must be sufficiently smooth and can satisfy the boundary conditions at four edges, which means they need to be third-order derivable and continuous at any point of the plate. With this in mind, the supplementary functions are chosen to be:

7a $$\begin{gathered} \xi_{a}^{1} (x) = \frac{9a}{{4\pi }}\sin \left( {\frac{\pi x}{{2a}}} \right) - \frac{a}{12\pi }\sin \left( {\frac{3\pi x}{{2a}}} \right), \hfill \\ \xi_{a}^{2} (x) = - \frac{9a}{{4\pi }}\cos \left( {\frac{\pi x}{{2a}}} \right) - \frac{a}{12\pi }\cos \left( {\frac{3\pi x}{{2a}}} \right), \hfill \\ \xi_{a}^{3} (x) = \frac{{a^{3} }}{{\pi^{3} }}\sin \left( {\frac{\pi x}{{2a}}} \right) - \frac{{a^{3} }}{{3\pi^{3} }}\sin \left( {\frac{3\pi x}{{2a}}} \right), \hfill \\ \xi_{a}^{4} (x) = - \frac{{a^{3} }}{{\pi^{3} }}\cos \left( {\frac{\pi x}{{2a}}} \right) - \frac{{a^{3} }}{{3\pi^{3} }}\cos \left( {\frac{3\pi x}{{2a}}} \right), \hfill \\ \end{gathered}$$

7b $$\begin{gathered} \xi_{b}^{1} (y) = \frac{9b}{{4\pi }}\sin \left( {\frac{\pi y}{{2b}}} \right) - \frac{b}{12\pi }\sin \left( {\frac{3\pi y}{{2b}}} \right), \hfill \\ \xi_{b}^{2} (y) = - \frac{9b}{{4\pi }}\cos \left( {\frac{\pi y}{{2b}}} \right) - \frac{b}{12\pi }\cos \left( {\frac{3\pi y}{{2b}}} \right), \hfill \\ \xi_{b}^{3} (y) = \frac{{b^{3} }}{{\pi^{3} }}\sin \left( {\frac{\pi y}{{2b}}} \right) - \frac{{b^{3} }}{{3\pi^{3} }}\sin \left( {\frac{3\pi y}{{2b}}} \right), \hfill \\ \xi_{b}^{4} (y) = - \frac{{b^{3} }}{{\pi^{3} }}\cos \left( {\frac{\pi y}{{2b}}} \right) - \frac{{b^{3} }}{{3\pi^{3} }}\cos \left( {\frac{3\pi y}{{2b}}} \right). \hfill \\ \end{gathered}$$

Based on Eq. (7a) and (7b), it is easy to obtain the following form:

8a $$\begin{gathered} \frac{{{\text{d}}\xi_{a}^{1} (0)}}{{{\text{d}}x}} = 1,\frac{{{\text{d}}\xi_{a}^{2} (0)}}{{{\text{d}}x}} = 0,\frac{{{\text{d}}\xi_{a}^{3} (0)}}{{{\text{d}}x}} = 0,\frac{{{\text{d}}\xi_{a}^{4} (0)}}{{{\text{d}}x}} = 0; \hfill \\ \frac{{{\text{d}}^{3} \xi_{a}^{1} (0)}}{{{\text{d}}x^{3} }} = 0,\frac{{{\text{d}}^{3} \xi_{a}^{2} (0)}}{{{\text{d}}x^{3} }} = 0,\frac{{{\text{d}}^{3} \xi_{a}^{3} (0)}}{{{\text{d}}x^{3} }} = 1,\frac{{{\text{d}}^{3} \xi_{a}^{4} (0)}}{{{\text{d}}x^{3} }} = 0; \hfill \\ \frac{{{\text{d}}\xi_{a}^{1} (a)}}{{{\text{d}}x}} = 0,\frac{{{\text{d}}\xi_{a}^{2} (a)}}{{{\text{d}}x}} = 1,\frac{{{\text{d}}\xi_{a}^{3} (a)}}{{{\text{d}}x}} = 0,\frac{{{\text{d}}\xi_{a}^{4} (a)}}{{{\text{d}}x}} = 0; \hfill \\ \frac{{{\text{d}}^{3} \xi_{a}^{1} (a)}}{{{\text{d}}x^{3} }} = 0,\frac{{{\text{d}}^{3} \xi_{a}^{2} (a)}}{{{\text{d}}x^{3} }} = 0,\frac{{{\text{d}}^{3} \xi_{a}^{3} (a)}}{{{\text{d}}x^{3} }} = 0,\frac{{{\text{d}}^{3} \xi_{a}^{4} (a)}}{{{\text{d}}x^{3} }} = 1. \hfill \\ \end{gathered}$$

8b $$\begin{gathered} \frac{{{\text{d}}\xi_{b}^{1} (0)}}{{{\text{d}}y}} = 1,\frac{{{\text{d}}\xi_{b}^{2} (0)}}{{{\text{d}}y}} = 0,\frac{{{\text{d}}\xi_{b}^{3} (0)}}{{{\text{d}}y}} = 0,\frac{{{\text{d}}\xi_{b}^{4} (0)}}{{{\text{d}}y}} = 0; \hfill \\ \frac{{{\text{d}}^{3} \xi_{b}^{1} (0)}}{{{\text{d}}y^{3} }} = 0,\frac{{{\text{d}}^{3} \xi_{b}^{2} (0)}}{{{\text{d}}y^{3} }} = 0,\frac{{{\text{d}}^{3} \xi_{b}^{3} (0)}}{{{\text{d}}y^{3} }} = 1,\frac{{{\text{d}}^{3} \xi_{b}^{4} (0)}}{{{\text{d}}y^{3} }} = 0; \hfill \\ \frac{{{\text{d}}\xi_{b}^{1} (b)}}{{{\text{d}}y}} = 0,\frac{{{\text{d}}\xi_{b}^{2} (b)}}{{{\text{d}}y}} = 1,\frac{{{\text{d}}\xi_{b}^{3} (b)}}{{{\text{d}}y}} = 0,\frac{{{\text{d}}\xi_{b}^{4} (b)}}{{{\text{d}}y}} = 0; \hfill \\ \frac{{{\text{d}}^{3} \xi_{b}^{1} (b)}}{{{\text{d}}y^{3} }} = 0,\frac{{{\text{d}}^{3} \xi_{b}^{2} (b)}}{{{\text{d}}y^{3} }} = 0,\frac{{{\text{d}}^{3} \xi_{b}^{3} (b)}}{{{\text{d}}y^{3} }} = 0,\frac{{{\text{d}}^{3} \xi_{b}^{4} (b)}}{{{\text{d}}y^{3} }} = 1. \hfill \\ \end{gathered}$$

These conditions show that each function of Eq. (7a) and (7b) represents either the first or the third derivative of the transverse displacement function at one of the edges [32]. This will guarantee that the two-dimensional Fourier series in Eq. (6) have at least three continuous derivatives for $\forall (x,y) \in ([0,a] \otimes [0,b])$.

The potential energy of the Kirchhoff plate model due to bending is equal to:

9 $$\begin{aligned} V_{{\text{b}}} = & \frac{1}{2}\iint {D\left[ {\left( {\frac{{\partial^{2} w}}{{\partial x^{2} }}} \right)^{2} + \left( {\frac{{\partial^{2} w}}{{\partial y^{2} }}} \right)^{2} + 2\mu \frac{{\partial^{2} w}}{{\partial x^{2} }}\frac{{\partial^{2} w}}{{\partial y^{2} }} + 2(1 - \mu )\left( {\frac{{\partial^{2} w}}{\partial x\partial y}} \right)^{2} } \right]}{\text{d}}x{\text{d}}y \\ & - \frac{1}{2}\iint {\frac{E\alpha h\Delta T}{{1 - \mu }}\left[ {\left( {\frac{\partial w}{{\partial x}}} \right)^{2} + \left( {\frac{\partial w}{{\partial y}}} \right)^{2} } \right]}{\text{d}}x{\text{d}}y \\ \end{aligned}$$

The potential energy of constraints at all edges is equal to:

10 $$\begin{aligned} V_{{\text{s}}} = & \frac{1}{2}\int_{0}^{a} {\left[ {k_{y0} w^{2} + K_{y0} \left( {\frac{\partial w}{{\partial y}}} \right)^{2} } \right]}_{y = 0} {\text{d}}x + \frac{1}{2}\int_{0}^{a} {\left[ {k_{yb} w^{2} + K_{yb} \left( {\frac{\partial w}{{\partial y}}} \right)^{2} } \right]}_{y = b} {\text{d}}x \\ & + \frac{1}{2}\int_{0}^{b} {\left[ {k_{x0} w^{2} + K_{x0} \left( {\frac{\partial w}{{\partial x}}} \right)^{2} } \right]}_{x = 0} {\text{d}}y + \frac{1}{2}\int_{0}^{b} {\left[ {k_{xa} w^{2} + K_{xa} \left( {\frac{\partial w}{{\partial x}}} \right)^{2} } \right]}_{x = a} {\text{d}}y \\ \end{aligned}$$

where *k*_*x*0_,* k*_*xa*_,* k*_*y*0_ and* k*_*yb*_ represent the linear spring constants at *x* = 0, *x* = *a*, *y* = 0 and *y* = *b*, *K*_*x*0_, *K*_*xa*_, *K*_*y*0_ and *K*_*yb*_ represent the rotational spring constants at *x* = 0, *x* = *a*, *y* = 0 and *y* = *b*, respectively. For a free edge, the stiffness for the transverse spring and the rotational spring is set as 0. For a clamped edge, the stiffness for the transverse spring and the rotational spring is infinitely large. However, to avoid the issue of ill-conditioned matrix in numerical calculations, the stiffness of the transverse spring and the rotational spring for the clamped edge is set to 10^9^. The kinetic energy due to lateral displacement is given by

11 $$T = \frac{1}{2}\iint {\int_{ - h/2}^{h/2} \rho }\dot{w}^{2} dzdxdy = \frac{1}{2}\iint {\rho h\dot{w}^{2} }dxdy$$

Then, the Lagrangian is expressed as

12 $$L = V_{{\text{b}}} + V_{{\text{s}}} - T$$

According to the Hamilton's principle,

13 $$\frac{\partial L}{{\partial A_{mn} }} = 0,\frac{\partial L}{{\partial c_{m}^{l} }} = 0,\frac{\partial L}{{\partial d_{n}^{l} }} = 0.$$

Taking Eq. (6) - (12) into Eq. (13), after simplifying, we have the following matrix equation:

14 $$\left( {{\mathbf{K}} - \omega^{2} {\mathbf{M}}} \right){\mathbf{a}} = 0$$

where **K** is the stiffness matrix and **M** is the mass matrix.

By solving the corresponding characteristic equation of Eq. (14), the eigenvalues and eigenvectors can be obtained as well as a series of natural frequencies *ω* and corresponding vectors **a**.

### Atomistic simulation

To investigate the vibrational properties of the diamane, MD simulations have been performed on a nanoplate (as shown in Fig. 1a) with a length of 7.9 nm and a width of 8.1 nm. For describing the C–C and C–H atomic interactions, the widely used adaptive intermolecular reactive empirical bond order (AIREBO) potential was employed [33]. This potential contains short-range interactions, long range van der Waals interactions and dihedral terms, which has been shown to well represent the binding energy and elastic properties of carbon materials. [34–36]. Previous studies have shown that AIREBO potential can reproduce well the elastic properties of carbon systems, such as CNT [37–39] and graphene [40–43]. To further confirm the effectiveness of AIREBO potential, some test simulations have been conducted on the diamane nanosheet. The results (see Supporting Information S1 in Additional file 1) show that the AIREBO potential is suitable for the MD simulation of diamane.

The diamane sample was relaxed to a minimum energy state using the conjugate gradient energy minimization [44]. After the energy minimization, the corresponding boundary conditions were applied. As illustrated in Fig. 1a, 0.15 nm wide from the edge of the sample (shown as the blue region) was defined as the boundary. For the clamped boundary condition, the atoms in this region were fixed to their initial positions and their velocities were set to 0 during the whole simulation. Then, the Nose–Hoover thermostat [44, 45] was employed to equilibrate the unfixed structure oscillating thermally for 4 ns. To obtain basic material parameters of diamane, uniaxial tension and pure bending deformation were done by MD simulations. A time step of 1 fs was used for all simulations, and non-periodic boundary conditions were applied in the all directions. Except for special instructions, all simulations are under 50 K. All MD simulations were performed using the open-source code Large-scale Atomic/Molecular Massively Parallel Simulator (LAMMPS) [46].

## Results and discussion

### Calibration of mechanical parameters

For the purpose of investigating the vibrations theoretically, basic mechanical parameters of diamane are needed. Considering the potential size effect of such ultrathin nanostructures, diamane’s static mechanical properties are determined by MD simulations. To determine the effective height and the effective Young’s modulus, tensile stiffness and bending stiffness are obtained from the tensile tests and pure bending tests respectively.

**Fig. 2:**
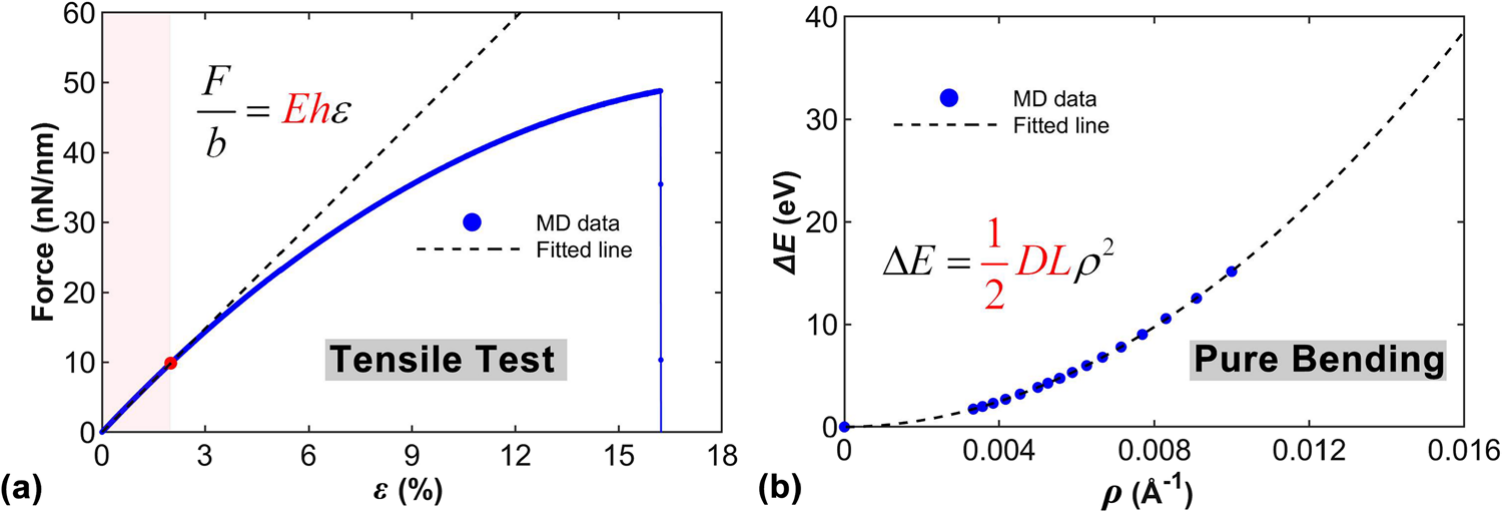
Tensile and pure bending tests of the diamane sample. **a** The force strain curve from the tensile tests. **b** The energy curvature curve from the pure bending tests

Through the uniaxial tension of diamane, the force-strain curve is obtained as shown in Fig. 2a. The detailed discussion about the tensile test is presented in Supplementary Information S2 in Additional file 1. Since the vibration considered in this work is within the linear elastic region of diamane, the data with the strain up to 2% is utilized to fit the tensile stiffness. The value is calibrated as 499.55 nN/nm (*Eh*). The Poisson’s ratio *μ* is also calculated as 0.06 based on the uniaxial tension simulation. Pure bending deformation is applied on a diamane sample with the size of 7.8 nm × 8.1 nm to access the bending stiffness. Following previous works on similar carbon materials [47, 48], different curvatures are imposed to bend the diamane structure. Then, the curved diamane is bounded to an idealized surface with the interactions being described by a Lennard–Jones (LJ) 9/3 potential expressed as $E_{wall} = \xi [2/15(\sigma /r)^{9} - (\sigma /r)^{3} ]$, where *ξ* and *σ* are chosen as 0.65 eV and 2 Å. Previous works [47, 48] have proved that the local strain on the sample due to the artificial surface is ignorable. The bending strain energy ∆*E* is estimated from ∆*E* = *E*_r_—*E*_0_, where *E*_r_ and *E*_0_ are the potential energies of bent sample and initial flat sample respectively. Figure 2b shows the bending energy curvature curve. Based on the nonlinear least square fitting, the bending stiffness *D* is derived as 3788.74 eV·Å. Combining the tensile stiffness and the bending stiffness, the effective height of diamane is calculated as 4.24 Å and the effective Young’s modulus is determined as 1179 GPa.

**Fig. 3:**
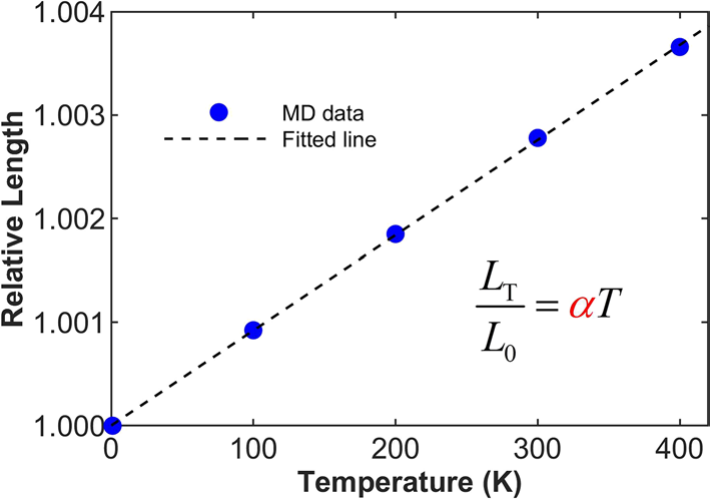
The relative lengths of diamane sheet under different temperatures for the calculation of the thermal expansion coefficient. The black dashed line is the fitted line based on the equation shown in the figure

To investigate the influence of thermal expansion, the diamane’s coefficient of thermal expansion is calculated. By MD simulations, the diamane sample is relaxed under different temperatures. Figure 3 plots the relative length, defined as the ratio of sample length under temperature *T* and 0 K, as a function of temperature *T*. By linear fitting, the coefficient of thermal expansion is determined as 9.17 × 10^–6^ K^−1^.

### Vibration of thin diamond

**Fig. 4:**
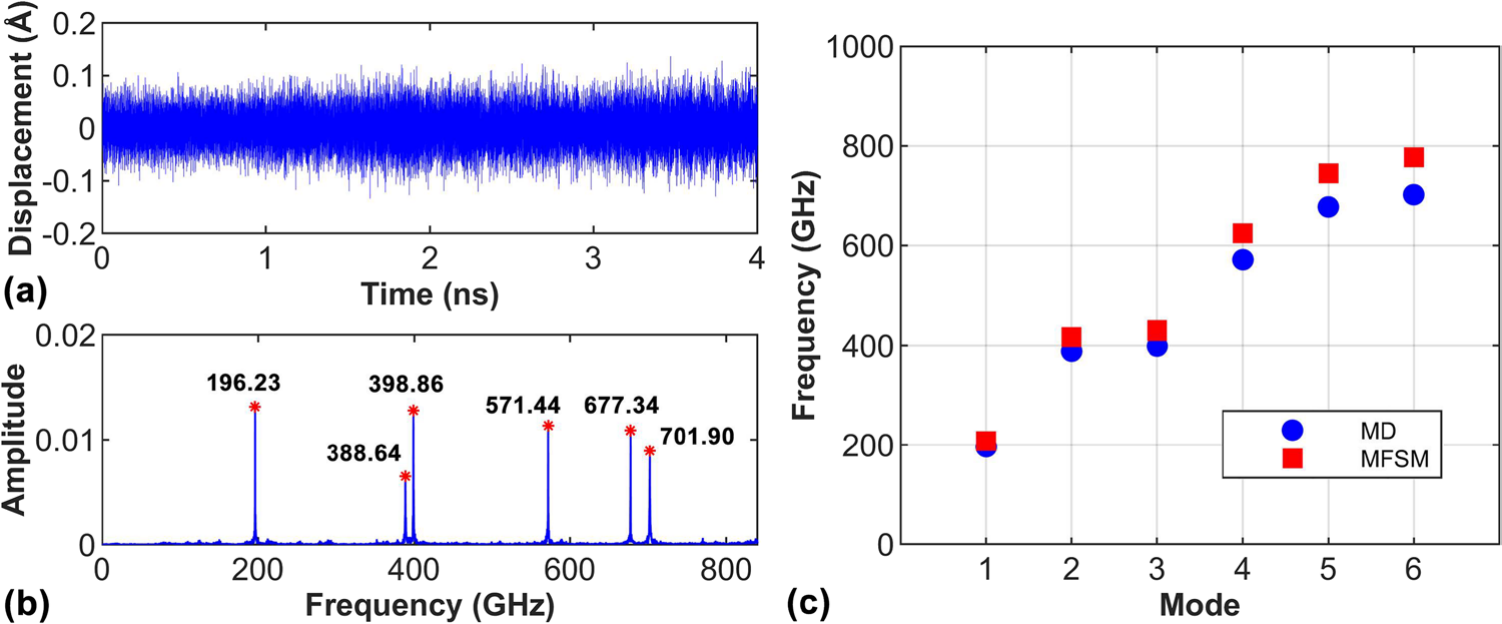
Thermally induced vibrations of diamane sample. **a** The time history of displacement of one carbon atom of the diamane. **b** The corresponding frequency spectrum obtained from FFT analysis. The first six order natural frequencies are marked in red asterisks. **c** The first six order natural frequencies obtained from MD simulations and predicted by the Kirchhoff plate model using MFSM

In this subsection, the vibration of diamane with all boundaries clamped is investigated. Figure 4a shows the time history of out-of-plane displacement of one carbon atom of the diamane from MD simulations. By applying the Fast Fourier transform (FFT) on the displacement data, the vibrational spectrum is obtained as illustrated in Fig. 4b and each peak represents one natural frequency of the diamane. The first six natural frequencies are identified in Fig. 4b and are marked with blue circular points in Fig. 4c. Using the MFSM, the natural frequencies predicted by the rectangle Kirchhoff plate model are plotted with red square points in Fig. 4c. It is found that, for the first three order natural frequencies, the differences between the Kirchhoff plate models and the MD simulation results are very small. However, the higher order frequencies predicted by the Kirchhoff plate models deviate from those obtained by MD simulations increasingly as shown in Fig. 4c. Such deviation is caused by the shear deformation in the thickness direction not being considered in the Kirchhoff plate model, which may play an important role in the high order frequencies. Overall, the rectangle Kirchhoff plate model can give a reasonable prediction to the natural frequencies of diamane sample obtained by MD simulations.

**Fig. 5:**
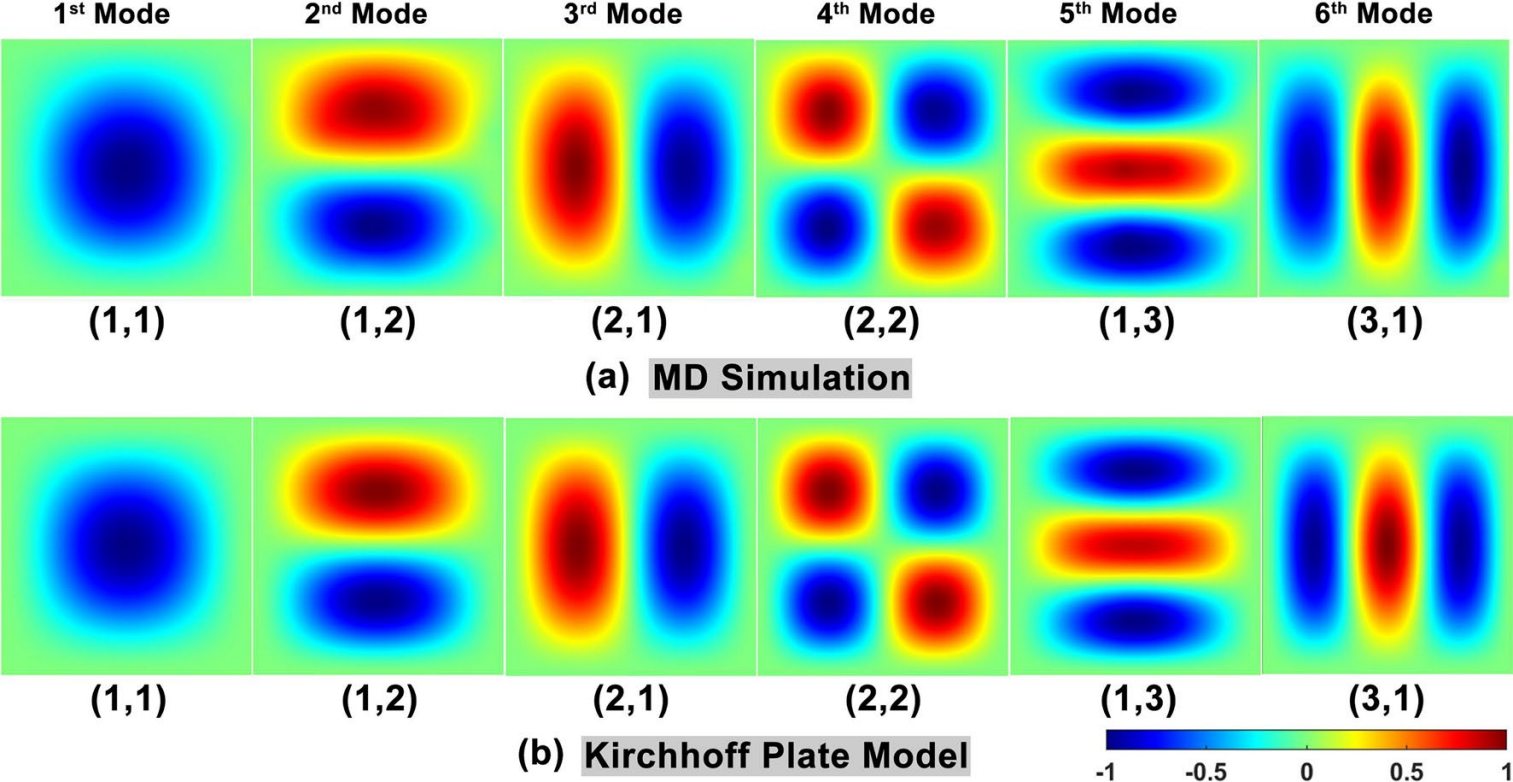
First six modal shapes of the diamane sample with all boundary clamped. **a** The normalized modal shapes are extracted from MD simulations. **b** The normalized modal shapes are predicted by the Kirchhoff plate model

Subsequently, the first six order modal shapes of diamane are studied. In order to extract the modal shapes from MD simulations, 64 carbon atoms are uniformly selected from the diamane sample (see Supporting Information S3 for details in Additional file 1). By applying the FFT on the displacement data of 64 carbon atoms, the modal shapes can be reconstructed based on the amplitude and phase information of corresponding natural frequencies. Figure 5a shows the normalized modal shapes from MD simulations. The modal shapes can also be marked as (*m*, *n*), where *m* and *n* are the half wavenumbers in the *x* and *y* directions of the diamane sample, respectively. The first six order modal shapes are recognized as (1, 1), (1, 2), (2, 1), (2, 2), (1, 3) and (3, 1). On the other hand, the modal shapes can be easily calculated by the rectangle Kirchhoff plate model using the MFSM. Figure 5b plots the normalized modal shapes predicted by the Kirchhoff plate model. It is clear that the modal shapes of the Kirchhoff plate model are in good agreement with those from MD simulations, which further confirms that the rectangle Kirchhoff plate model can well describe the vibrational properties of diamane with all boundaries clamped.

### Boundary conditions influence

**Fig. 6:**
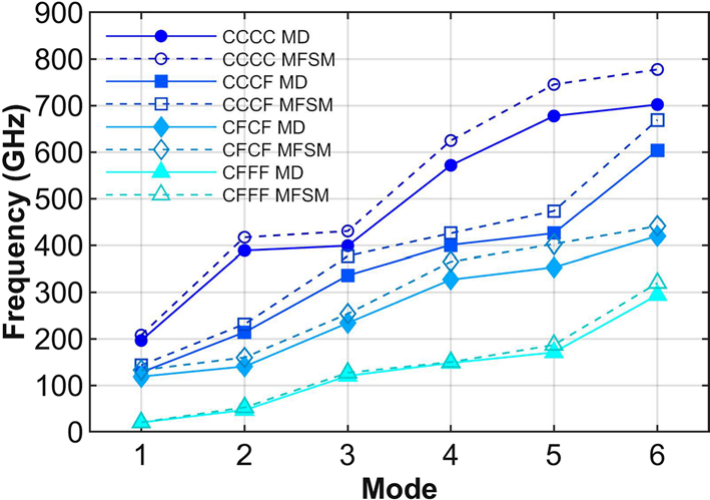
The natural frequencies of diamane sample under different boundary conditions. The solid lines are obtained from MD simulations, while the dashed lines are predicted by the Kirchhoff plate model using MFSM

The vibrational properties of the diamane under different boundary conditions are studied in this subsection. Four different boundary conditions including CCCC, CCCF, CFCF and CFFF shown in Fig. 1b are considered in both MD simulations and rectangle Kirchhoff plate models. In MD simulations, these different boundary conditions can be realized by applying different constraints to corresponding boundaries. For the rectangle Kirchhoff plate models, different boundary conditions can be achieved by setting the stiffness constants of springs in MFSM. Figure 6 presents the first six order natural frequencies of the diamane under different boundary conditions predicted by MD simulations shown in solid markers and Kirchhoff plate models shown in hollow markers. As above, it is found that the deviation of the natural frequencies between MD simulations and Kirchhoff plate models grows with the order. Such deviation is also resulted from the shear deformation not being considered in the Kirchhoff plate model. For the diamane sample under different boundary conditions, the rectangle Kirchhoff plate model can reasonably predict the natural frequencies obtained by MD simulations.

**Fig. 7:**
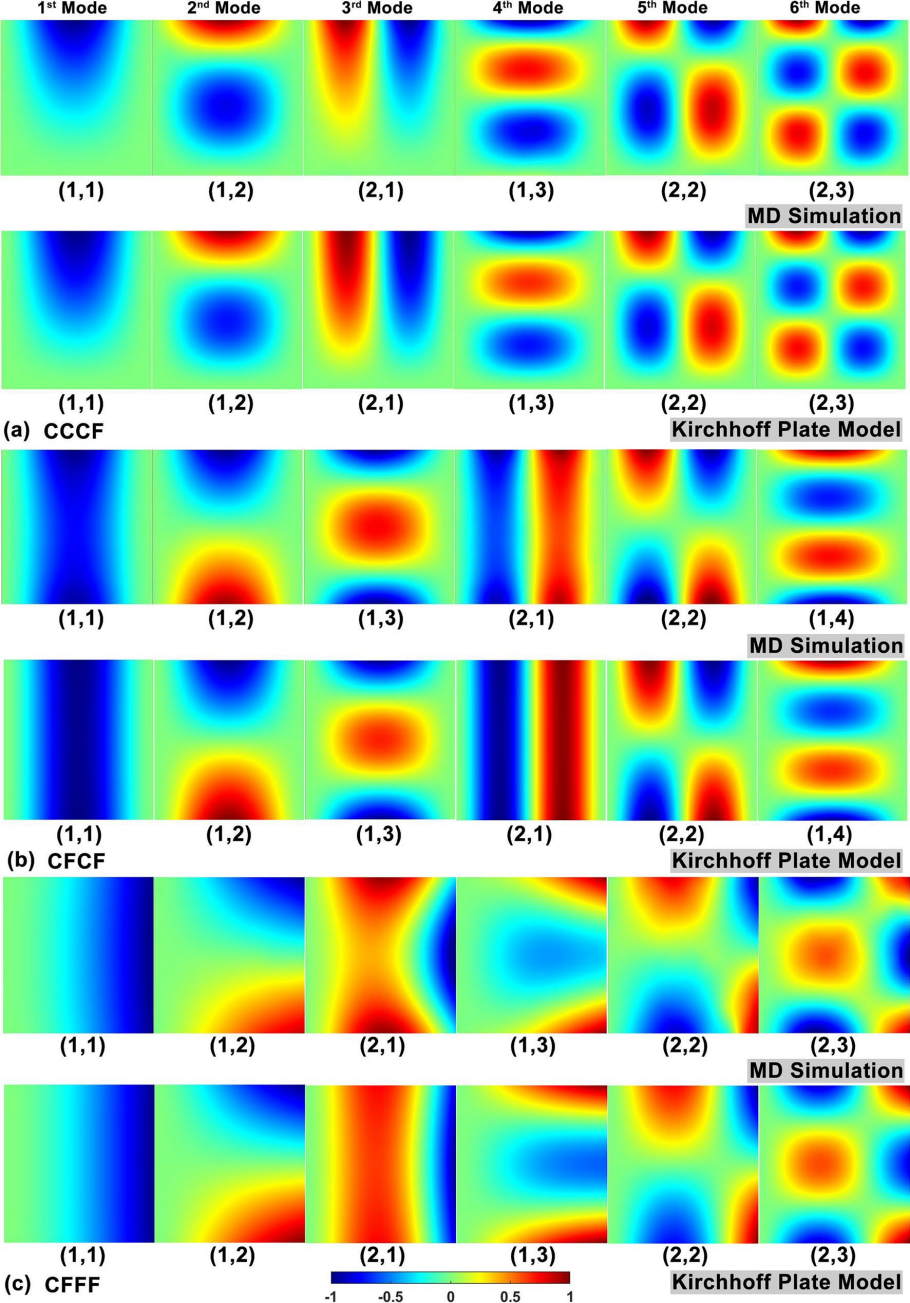
First six modal shapes of the diamane sample extracted from MD simulations and predicted by the Kirchhoff plate model under different boundary conditions: **a** CCCF; **b** CFCF and **c** CFFF

For further information on the boundary conditions influence, the corresponding modal shapes of diamane’s vibration are studied. As above, the normalized modal shapes can be obtained from MD simulations and predicted by Kirchhoff plate models as illustrated in Fig. 7. The modal shapes under boundary conditions of CCCF, CFCF and CFFF are presented in Fig. 7a, 7b and 7c, respectively. It is clear that, for all boundary conditions considered in this work, the modal shapes obtained from MD simulations have the same half wavenumbers in the *x* and *y* directions to those predicted by Kirchhoff plate models. By comparing details, there are some small differences between certain modal shapes pairs, such as the 1^st^ and 3^rd^ modes in CCCF, 1^st^ and 4^th^ modes in CFCF, 3^rd^ and 4^th^ modes in CFFF. It is found that such differences are related to free boundaries, which indicates that the edge effect may influence the modal shapes. Previous work [22] has shown that the flat free edge of diamane has little effect on its vibrational properties compared to warped free edge of other two-dimensional materials like graphene. Hence, the rectangle Kirchhoff plate model can reasonably describe the vibrational properties of diamane under different boundary conditions.

### Temperature influence

Temperature is known as a significant factor which may affect the natural frequencies of nanomaterials’ vibrations. Here, we investigate the temperature influence to the diamane with all boundaries clamped by taking the thermal expansion into the consideration. In MD simulations, all boundaries of the diamane sample are clamped after the energy minimization. Then, different temperatures are applied. For the rectangle Kirchhoff plate models, the effect of the thermal expansion is considered in Eq. (1).

**Fig. 8:**
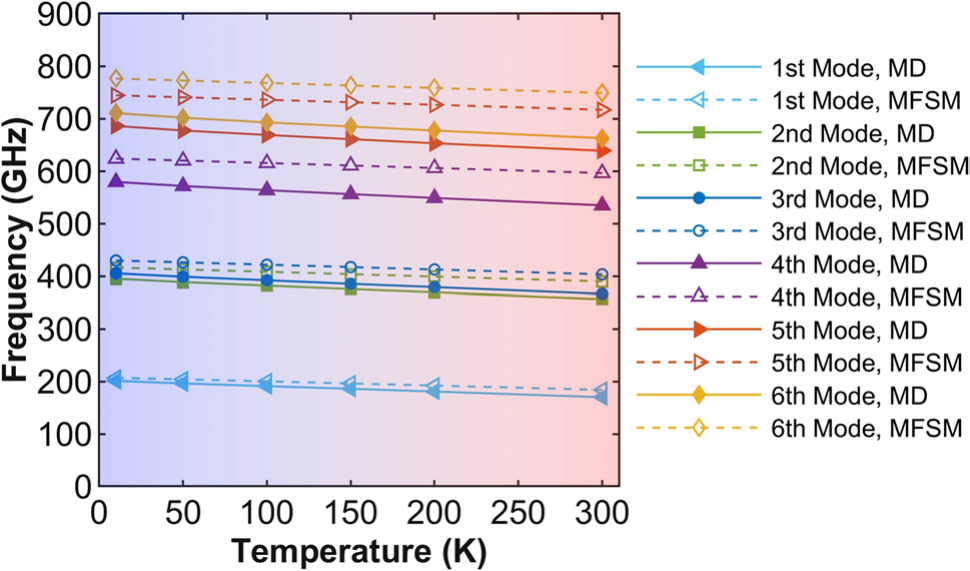
The natural frequencies of diamane sample different temperatures. The solid lines are obtained from MD simulations, while the dashed lines are predicted by the Kirchhoff plate model using MFSM

Figure 8 shows the first six natural frequencies of the diamane sample under different temperatures. Solid markers are the natural frequencies obtained from MD simulations, while hollow markers present the natural frequencies predicted by the Kirchhoff plate models described by Eq. (1). From the Fig. 8, it is clear to see that the natural frequencies decrease linearly and slightly with the increase of the temperature. Besides the deviation resulted from the shear deformation not being considered in the Kirchhoff plate model, the trends of natural frequencies obtained from MD simulations and predicted by Kirchhoff plate models are the same. Hence, the rectangle Kirchhoff plate model considering the thermal expansion can give a good prediction of the temperature influence to the natural frequencies for vibration of diamane.

## Conclusion

The MD simulation and Kirchhoff plate model were utilized to investigate the vibrational characteristics of the diamane sheet. The natural frequencies and corresponding modal shapes of the rectangular diamane sheet were obtained from both the MD simulations and Kirchhoff plate models. The results reveal that the differences in frequencies between the MD simulation and Kirchhoff plate model increase slightly with higher vibration orders. Such deviation is anticipated as the shear deformation is not accounted for in the Kirchhoff plate model. Moreover, it was observed that the free boundary condition has a minor impact on the modal shapes, suggesting that the modal shapes may be influenced by edge effects. As the temperature rises, the natural frequencies of the clamped diamane sheet decrease, a phenomenon well described by the Kirchhoff plate model considering thermal expansion. In summary, this study demonstrates that the rectangular Kirchhoff plate model can reasonably predict the vibration properties of diamane sheets, thus shedding light on their potential applications in nanoscale mechanical resonators.

### Supplementary Information

**Additional file 1**. Supplementary material

## Data Availability

All data generated or analyzed during this study are included in this published article.
