# Supplementary material for: Analysis of the vibrational characteristics of diamane nanosheet based on the Kirchhoff plate model and atomistic simulations
Source: Discov Nano. 2023 Aug 31;18(1):108. doi: 10.1186/s11671-023-03887-5 (PMC10471560; doi:10.1186/s11671-023-03887-5)
Supplement: Supplementary file 1 — Additional file 1. Supplementary material [file 11671_2023_3887_MOESM1_ESM.docx]

**Supporting Information**

**Analysis of the Vibrational Characteristics of Diamane Nanosheet Based on the Kirchhoff Plate Model and Atomistic Simulations**

*Zhuoqun Zheng^1^, Fengyu Deng^1^, Zhu Su^1^, Haifei Zhan^2,3^, Lifeng Wang^1,^*^[[1]](#footnote-1)^*

*^1^* *State Key Laboratory of Mechanics and Control for Aerospace Structures, Nanjing University of Aeronautics and Astronautics, Nanjing, 210016, China*

*^2^ College of Civil Engineering and Architecture, Zhejiang University, Hangzhou, 310058, China*

*^3^ School of Mechanical, Medical and Process Engineering, Queensland University of Technology, Brisbane QLD 4001, Australia*

**S1. Effectiveness of AIREBO potential for diamane nanosheet**

To examine the effectiveness of the AIREBO potential, two stages of simulations are performed. Firstly, the energy minimization for the diamane nanosheet under periodic boundary condition is conducted. Figure S1a plots the total energy of the system as a function of steps. From the system energy curve, it is found that the total energy decreases quickly in the beginning and then becomes stable. Furthermore, the radial distribution function (RDF) profile in Figure S1b indicates that the C-H bonds are 1.09 Å and C-C bonds are 1.54 Å. According to the atomic configuration from the DFT calculation [1], the C-H bonds are 1.10 Å and the C-C bonds are 1.52 Å. These results are in a good agreement. Secondly, the structural relaxation under 50 K is conducted on the diamane nanosheet for 500 ps under the NPT ensemble. As shown in Figure S1c, the system energy fluctuates in a very small range during the last 400 ps. Additionally, the RDF profile shows the bond lengths of C-H and C-C bonds stay in a reasonable range. It means that the diamane nanosheet can well maintains its structure in the MD simulations using the AIREBO potential. Overall, based on these tests, the AIREBO potential is appropriate for the MD simulation of diamane nanosheet.


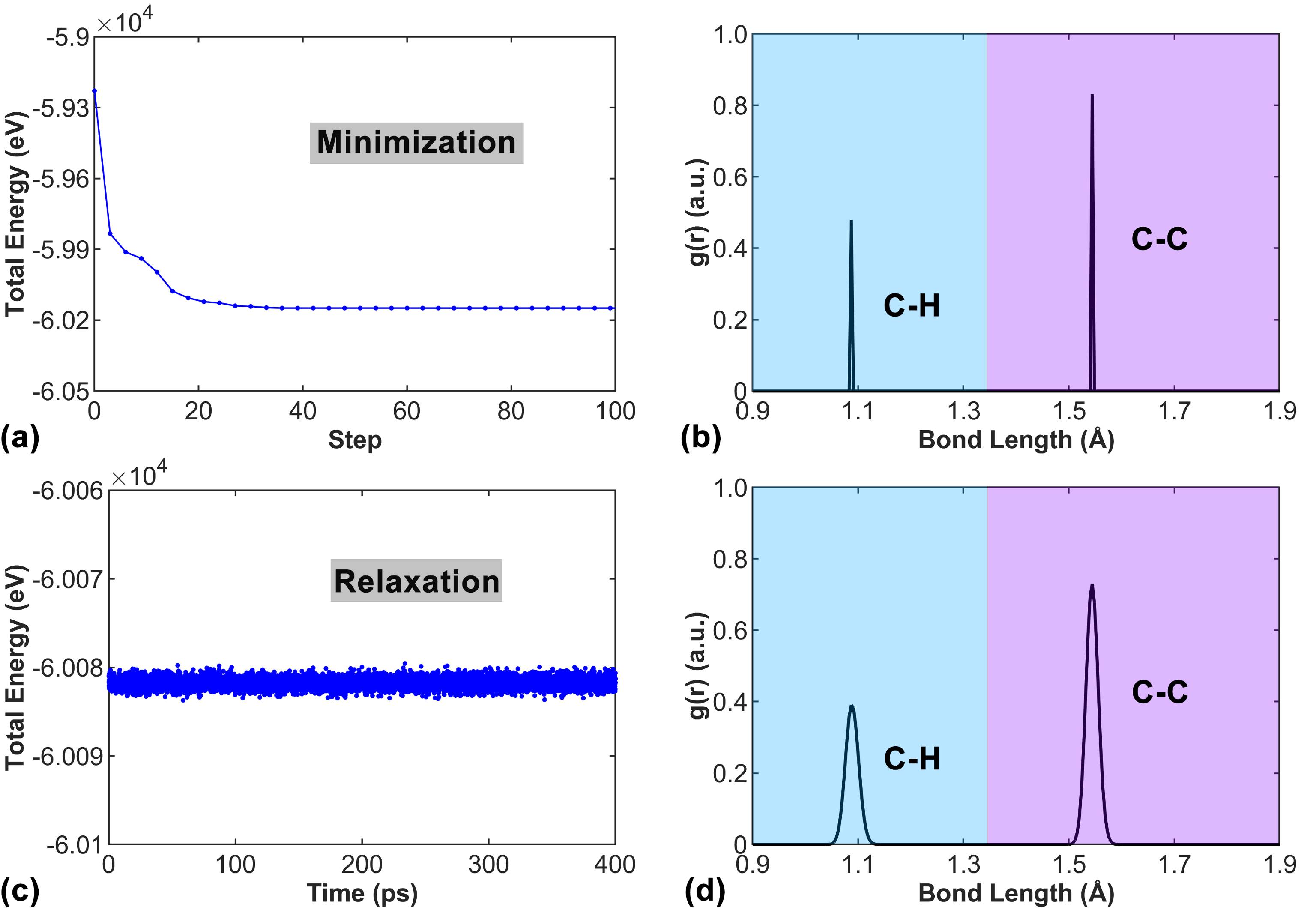


**Figure S1** Test simulations of the AIREBO potential for diamane. (a) The system energy curve during the energy minimization; (b) The RDF profile of the diamane structure after the energy minimization; (c) The system energy during the last 400 ps of relaxation simulation; (d) The RDF profile of the diamane structure during the last 400 ps of relaxation simulation.

**S2. Tensile tests of diamane**

The uniaxial tension simulations have been conducted on the diamane sheet under 50 K. Figure S2a presents the tensile force as a function of strain. It is found that, when the strain passes a certain value (16.22%), the force experiences a sudden decrease. It indicates that the diamane has a brittle behavior. To further confirm this phenomenon, the snapshots at the tensile strain of 0%, 16.20% and 16.25% are presented in Figures S2b-d. They are colored based on the atomic stress in tensile direction. The brittle characteristic can also be found from the snapshots.


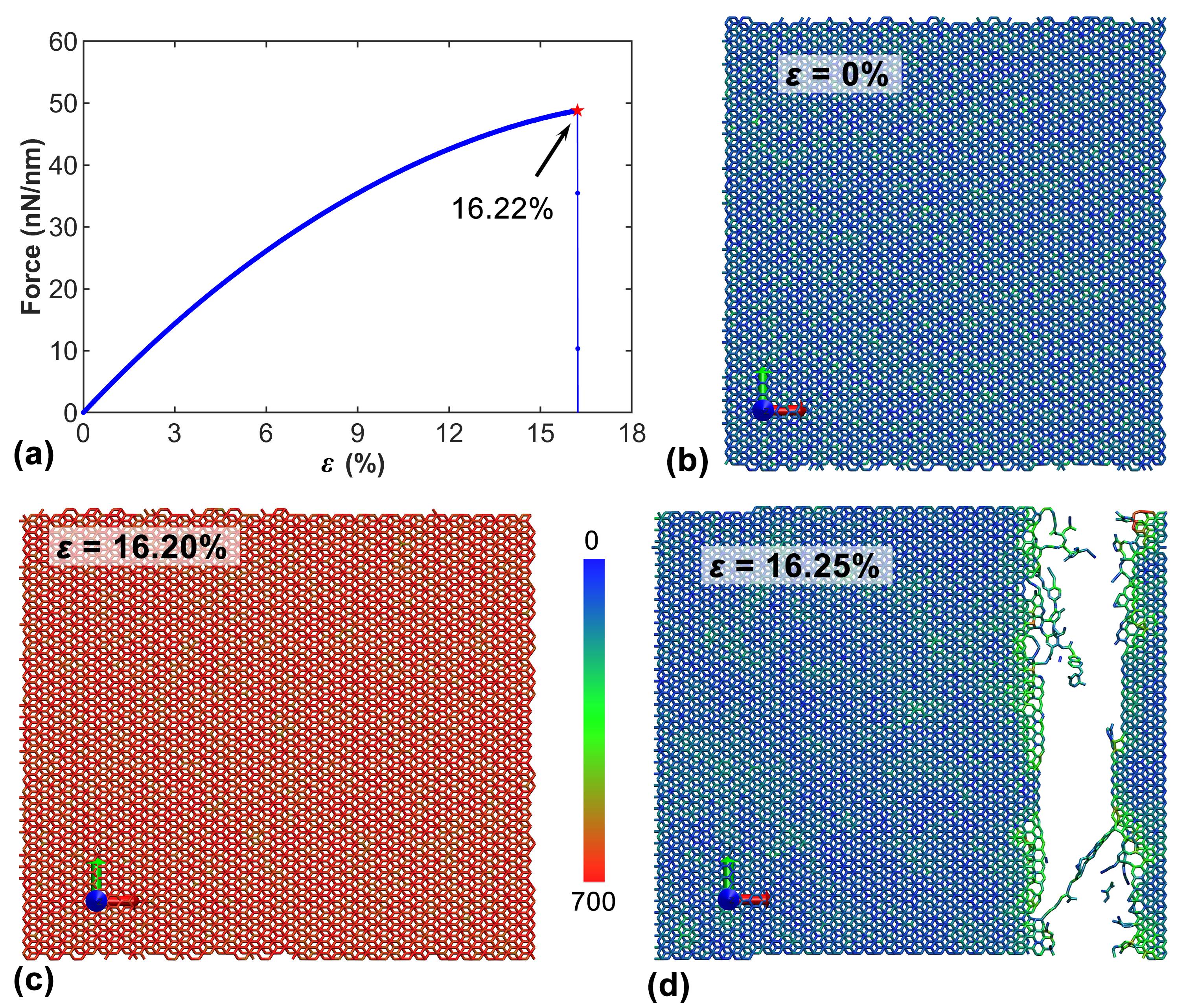


**Figure S2.** Tensile deformation of the diamane sheet: (a) Tensile force as a function of strain. The snapshots at the tensile strain of (b) 0%, (c) 16.20% and (d) 16.25%. Atoms are colored based on the atomic stress in tensile direction.

**S3. The modal shapes from MD simulations**

In order to extract the modal shapes from MD simulations, 64 carbon atoms are uniformly selected from the diamane sample. As illustrated in **Figure S3**, these 64 carbon atoms are uniformly distributed on the diamane sheet and can form a set of grids. The detailed amplitude and phase information of corresponding natural frequencies can be obtained by applying the FFT on the displacement data from MD simulations. Finally, the modal shapes can be reconstructed.


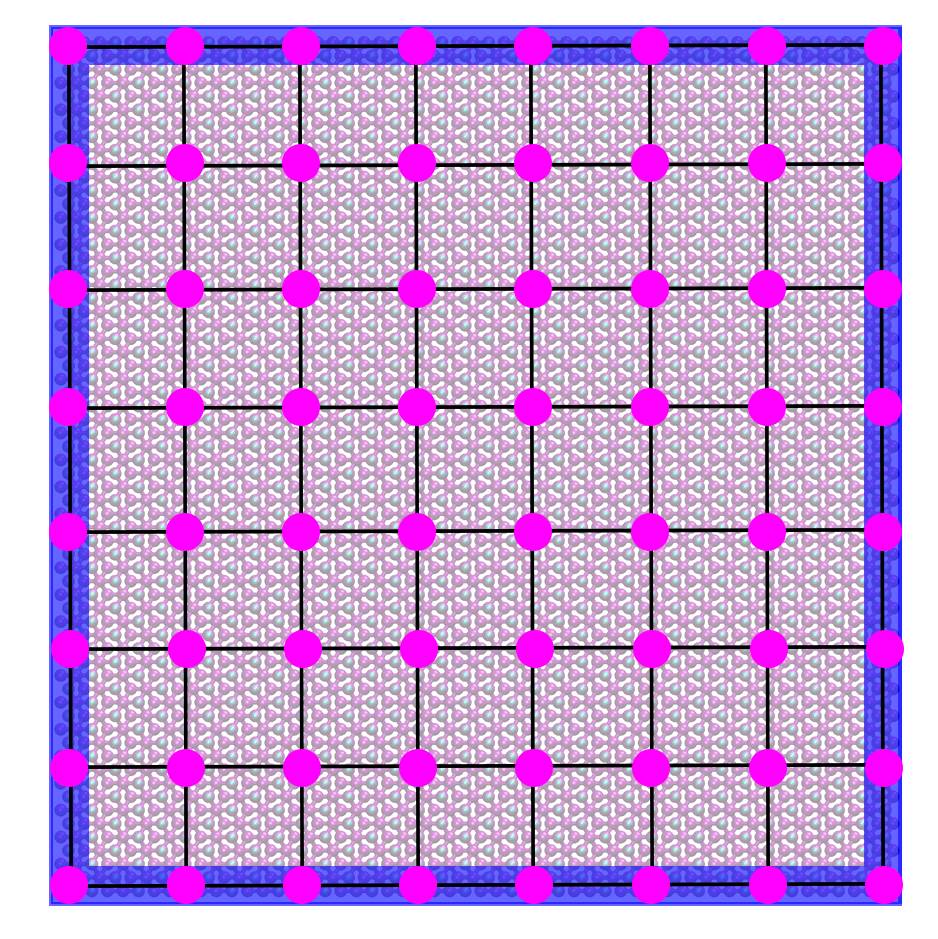


**Figure S3.** The diagram of 64 atoms selected from the diamane sheet.

**Reference**

[1] L.A. Chernozatonskii, P.B. Sorokin, A.G.e. Kvashnin, D.G.e. Kvashnin, Diamond-like C 2 H nanolayer, diamane: Simulation of the structure and properties, JETP Lett., 90 (2009) 134-138.

1. *Corresponding author. Email: [walfe@nuaa.edu.cn](mailto:walfe@nuaa.edu.cn) (Lifeng Wang) [↑](#footnote-ref-1)
